# ATG9A modulated by miR-195-5p can boost the malignant progression of cervical cancer cells

**DOI:** 10.1080/15592294.2023.2257538

**Published:** 2023-10-02

**Authors:** Xiaomin Liu, Zhen Liu, Yonggang Liu, Ning Wang

**Affiliations:** a Department of Gynecology, Chifeng Municipal Hospital, Chifeng Clinical Medical School of Inner Mongolia Medical University, Chifeng, China; b Department of Gynecology, The Second Hospital of Dalian Medical University, DaLian, China

**Keywords:** Autophagy, apoptosis, cervical cancer, miR-195-5p, ATG9A

## Abstract

Cervical cancer (CC) is a major public health problem, and its molecular mechanism requires further investigation. The goal of this study was to determine the role of miR-195-5p and the autophagy-related protein ATG9A in tumour metastasis, epithelial – mesenchymal transition (EMT), apoptosis, and autophagy of CC cells. Using bioinformatics analysis, we predicted ATG9A as a downstream target gene of miR-195-5p, an integral membrane protein required for autophagosome formation and involved in tumorigenesis. Next, western blotting and Quantitative Real-Time Polymerase Chain Reaction (qRT-PCR) showed that upregulation of miR-195-5p decreased protein and mRNA expression of ATG9A, and downregulation of miR-195-5p promoted ATG9A protein and mRNA expression. In addition, detection of the dual luciferase reporter gene further indicated ATG9A is a direct downstream target gene of miR-195-5p. Finally, the effects of miR-195-5p and ATG9A on CC cell proliferation, migration, invasion, EMT, autophagy, and apoptosis were evaluated *in vitro*. Our results showed that upregulation of miR-195-5p not only inhibits proliferation, migration, and the EMT of CC cells, but also induces apoptosis and autophagy. Conversely, downregulation of miR-195-5p increased malignant metastasis and the EMT of CC cells, and inhibited apoptosis as well as autophagy. In addition, miR-195-5p targeted and negatively regulated ATG9A, and rescue experiments suggested that overexpression of ATG9A could partially abolish miR-195-5p-mediated suppression of CC cells. Our findings improve our understanding of the mechanism of action of miR-195-5p in the malignant behaviour of CC. miR-195-5p is likely to be a promising cancer suppressor gene, which provides clinical evidence for targeted therapy of CC.

## Background

Cervical cancer (CC) ranks as the fourth most common cancer among women and seventh overall [1]. In China, CC is now the second most common cancer and the third leading cause of cancer death among women aged 15 to 44, with 100,700 new cases and 26,400 deaths from CC in 2013 [2,3]. In recent years, popularization of human papillomavirus vaccine and implementation of a wide range of cytological screening measures for CC have significantly improved the diagnostic accuracy and survival rate, but the high mortality caused by recurrence and metastasis of CC remains a difficult issue [4]. As a result, it is imperative to seek out novel and accurate diagnosis and treatment strategies for CC patients.

MicroRNAs (miRNAs), single-stranded small molecule RNAs composed of 20–25 nucleotides, regulate gene expression by binding the 3’ untranslated region (3’-UTR) of target genes [5]. Importantly, accumulated evidence has suggested that dysregulated miRNAs can act not only as oncogenes but also as tumour suppressors in different tissues to regulate biological behaviours of cancer, such as cell growth, migration, invasiveness, autophagy, and chemoresistance [6–8]. Previous studies have shown that miR-195 can function as a tumour suppressor in colorectal cancer [9], oesophageal cancer [10], non-small cell lung cancer [11], triple-negative breast cancer (TBNC) [12], and CC [13,14]. Recent data have suggested that miR-195-5p expression is reduced in CC tissues and cell lines, and downregulation of miR-195-5p is associated with higher Federation of International of Gynecologists and Obstetrician stage, deep stromal invasion, and lymph node metastasis [14,15]. Despite this, the molecular mechanism of miR-195-5p in CC remains ambiguous. In this study, we identified ATG9A as a target gene of miR-195-5p using the miRNA prediction website. Among the core autophagy-associated (ATG) proteins, ATG9A, the only transmembrane protein required for progression of autophagy, is localized in the phagophore/pre-autophagosomal structure (PAS) [16,17]. Autophagy is a process of protein degradation, which satisfies the metabolism of the cell itself to achieve homoeostasis and organelle renewal. Paradoxically, both increased autophagy and inhibition of autophagy inhibit tumorigenesis or promote cancer cell survival [18]. In other words, the effects of autophagy in various stages of cancer development have not yet been confirmed and will require further exploration. ATG9A has been shown to be upregulated in human oral squamous cell carcinoma [19], ovarian cancer [20], and TBNC [21], and is associated with overall survival and progression-free survival. In addition, Dai et al. [20], Pang et al. [22], and Xu et al. [23] have demonstrated that miR-29b, miR-34a, and miR-29a directly target ATG9A in different cell lines. However, the role of ATG9A in CC remains unknown.

Here, we found that miR-195-5p plays an anti-oncogene role in CC. Specifically, its overexpression inhibits cell neoplastic migration, lymph node metastasis, and EMT, and promotes apoptosis and autophagy, by targeting ATG9A. Rescue experiments showed that ATG9A overexpression partially recovered the inhibition of miR-195-5p on CC cells. Thus, the miR-195-5p/ATG9A axis may also bring new ideas for targeted treatment of CC patients.

## Methods

### Human CC cell culture and transfection

HeLa and SiHa cells were obtained from the Shanghai Institute of Biochemistry and Cell Biology (Shanghai, China) and cultured in RPMI 1640 medium (BioInd, Kibbutz Beit Haemek, Israel) containing 10% foetal bovine serum (FBS, BioInd) and 1% penicillin-streptomycin solution (BioInd) at 37°C and 5% CO_2_. Chemosynthetic hsa-miR-195-5p mimics, mimics control, hsa-miR-195-5p inhibitor, inhibitor control, ATG9A overexpression plasmid, and ATG9A negative control were obtained from Shanghai GenePharma Co., Ltd. (Shanghai, China). HeLa and SiHa cells were seeded at 80%–90% density and transfected with Lipofectamine 3000 (Invitrogen, Carlsbad, CA, USA) according to the manufacturer’s instructions.

### Bioinformatics analysis

MiRwalk 3.0 (http://mirwalk.umm.uni-heidelberg.de/), Starbase2.0 (http://starbase.sysu.edu.cn/starbase2/index.php), and TargetScan (http://www.targetscan.org/vert_72/) were used to predict target genes of miR-195-5p according to the manufacturers’ instructions.

### Luciferase reporter assay

DNA segments of the ATG9A 3’-UTR with the predicted connecting site of miR-195-5p and its mutant sequence were cloned into the pmirGLO luciferase reporter vector (Promega, USA) to construct the wild-type and mutant plasmids pmirGLO-ATG9A-Luc-WT and pmirGLO-ATG9A-Luc-MUT, respectively. The groups were co-transfected into HeLa and SiHa cells. The dual-luciferase reporter assay system (Promega, USA) was used to measure luciferase activity and was normalized using Renilla luciferase activity according to the manufacturer’s instructions and as previously described [13,24].

### RNA isolation and quantitative real-time polymerase chain reaction (qRT-PCR)

Total RNA including miRNAs was isolated from HeLa and SiHa cells using an miRNeasy Mini Kit (Qiagen, Hilden, Germany). Then, the extracted miRNAs were added to *Escherichia coli* Poly(A) Polymerase Reaction Buffer (NEB, Ipswich, MA, USA) to reverse all miRNAs using the GoScriptTM Reverse Transcription System (Promega). Next, the GoScript™ Reverse Transcription System (Promega) was used to synthesize first-strand cDNA optimized for quantitative PCR amplification. Gotaq® qPCR Master Mix (Promega) was subsequently applied for qRT-PCR according to the manufacturer’s instructions and as previously described [13]. All primers were synthesized by Guangzhou RiboBio Co., Ltd. (Guangzhou, China). The primer sequences were as follows:miR-195-5p, F: 5’-GCGTAGCAGCACAGAAATATTGGC-3’ and R: 5’-CTGTCGTCGTAGAGCCAGGGAA-3’; U6, F: 5’-CTCGCTTCGGCAGCACA-3’ and R: 5’-AACGCTTCACGAATTTGCGT-3’; ATG9A, F: 5’-CTGCCCTTCCGTATTGCAC-3’ and R: 5’- CTCACGTTTGTGGATGCAGAT-3;’ and GAPDH, F: 5’-TGCACCACCAACTGCTTAGC-3 and R: 5’-GGCATGGACTGTGGTCATGAG-3.The relative expression levels of miR-195-5p and ATG9A were normalized with U6 and GAPDH using the 2−^∆∆Ct^ method.

### Western blot analysis

After transfection, 50–100 μl of RIPA buffer (Beyotime, Shanghai, China) was added to HeLa and SiHa cells to extract whole cell protein. The protein concentration was measured using a BCA protein quantitative kit (Beyotime). Following separation of 40 μg of protein by 8% SDS-PAGE, the protein was transferred onto a PVDF membrane (Millipore, Burlington, MA, USA) by wet transformation. Then, the PVDF membrane was incubated with 5% skimmed milk for 1 h. Next, specific antibodies against the target proteins were added and the membrane was incubated overnight at 4°C. The antibodies used were as follows: ATG9A, E-cadherin, Snail, Vimentin, caspase-3, LC3-II, P62, and Bax (Cell Signaling Technology, MA, USA); and VEGFA, MMP-2, MMP-9, and Bcl-2 (Abcam, MA, USA). Next, the membrane was incubated with secondary antibody labelled with horseradish peroxidase (Proteintech, Rosemont, IL, USA) for 1 h. Finally, the immunological complexes were developed by enhanced chemiluminescence (ECL, Hercules, CA, USA). At least three independent experiments were performed.

### Cell proliferation assay

Cell proliferation viability was detected by the MTT assay (Sigma-Aldrich, St. Louis, MO, USA). HeLa and SiHa cells (2 × 10^3^ cells/per well) were seeded in 96-well plates and incubated for 24, 48, 72, and 96 h. Then, the cells were cultured with serum-free medium mixing with 10 μl MTT at regular time intervals for 4 h. The supernatant was removed, 100 μl dimethyl sulphoxide was added to each well, and the absorbance at 490 nm was measured using a microplate reader (ThermoFisher Scientific, USA) to examine cell viability. Assays were performed in triplicate followed by statistical analysis. This method was described in a previous study [13].

### Transwell invasion assay

The Transwell invasion assay was performed using 24-well Transwell plates with polycarbonate membrane and 8.0 μm pores (Corning, USA). After 24 h of transfection, Matrigel (4.0 μg/μl, 60 μl) was added into Transwell chambers (Corning, NY, USA) and cultured for 2–3 h at 37°C for gel solidification. RPMI 1640 medium containing 10% FBS was added to the lower chambers and cells were seeded in the upper chambers with serum-free medium at a density of 4 × 10^3^ cells/per well. After 48 h, the migrated cells were fixed with 4% paraformaldehyde and stained with 0.1% crystal violet for 30 min. Migrating cell numbers were counted in five random fields under an inverted phase microscope (Nikon, Japan) at a magnification of 100× as previously described [13]. Assays were performed in triplicate followed by statistical analysis.

### Wound healing assay

When HeLa and SiHa cells reached 90% confluence, cells were transfected. After 24 h, a 200 µl sterile pipette was used to scratch the monolayer of cells. Cells were cultured in serum-free medium and photographed with an inverted microscope magnified 100× (Nikon, Japan) at 0 and 48 h after wound formation. Assays were performed in triplicate followed by statistical analysis.

### TUNEL assay

The TUNEL assay was performed using by an In Situ Cell Death Detection Kit and In situ cell death assay kit TMR red (Germany, Rocho). After transfection for 24 h, CC cells were plated at 1 × 10^4^ cells/per well in a Nunc Lab-Tek II Chamber Slide System (Thermo Fisher Scientific. Inc., USA), a removable polystyrene medium chamber with an 8-well configuration. The cell samples were then fixed with 4% paraformaldehyde and 60 µl of TUNEL reaction mixture was added. The samples were stained with DAPI and analysed directly under a fluorescence microscope (Nikon, Japan). DAPI- and TUNEL-positive spots were analysed using ImageJ software, and TUNEL/DAPI × 100% was calculated as the ratio of apoptotic cells.

### Statistical analysis

All data are presented as mean ± standard deviation and statistical analysis was performed using SPSS 17.0 software (SPSS Inc., Chicago, IL, USA). Differences between two groups were analysed using the Student’s *t*-test. Statistical significance was accepted when *P* < 0.05.

## Results

### ATG9A is a target gene of miR-195-5p in CC cells

**Figure 1.**
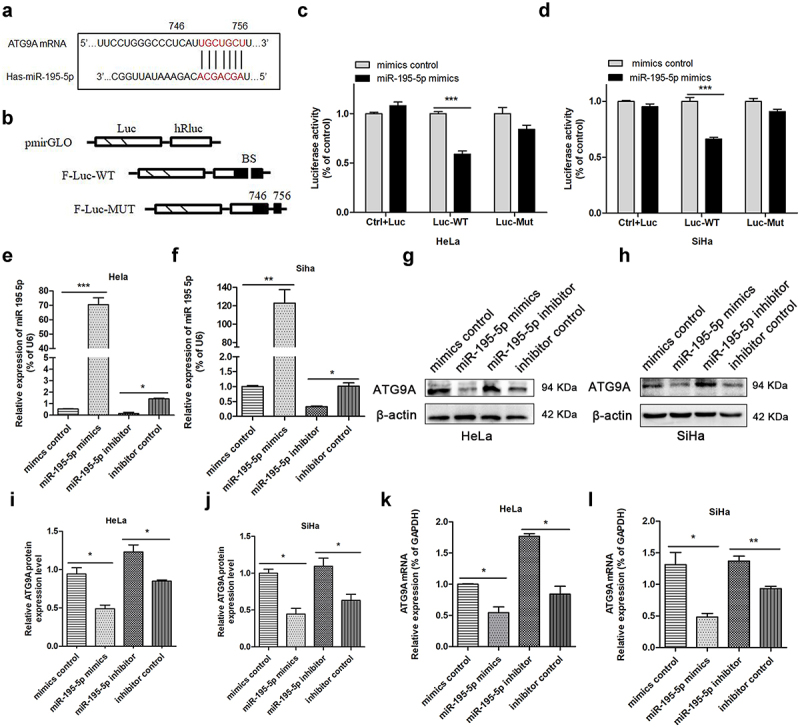
MiR-195-5p inhibits ATG9A translation. (a) targetscan, mirwalk, and starbase predicted 746–756 as the binding site of ATG9A and miR-195-5p. (b) wild type (WT) and mutant (MUT) ATG9A 3‘‑UTR luciferase reporter plasmids were constructed. (c,d) After transfection, luciferase activity was compared by double luciferase reporter assay. (e,f) miR-195-5p levels in HeLa and SiHa cells transfected with the mimics and inhibitor for 24 h. (g,h) After increasing or inhibiting the expression of miR-195-5p, expression of ATG9A was determined by western blotting (i,j) or qRT-PCR (k,l). Quantification is shown in the bar graph. Experiments were repeated three times (*n* = 3). Student’s *t*-tests were used for statistical analyses. *p < 0.05, **p < 0.01, and ***p < 0.001 versus control groups.

The potential molecular targets of miR-195-5p were predicted using miRwalk, Targetscan, and Starbase. ATG9A plays an important role in regulating tumour volume, regional lymph node involvement, and advanced stage in oral squamous cell carcinoma, TNBC, and ovarian cancer [19–21]. In addition, ATG9A is an important multi-spanning membrane protein in autophagy [16]. According to our bioinformatics analysis, there is a binding site between ATG9A and miR-195-5p (Figure 1(a)). We first constructed a double luciferase reporter vector to detect whether miR-195-5p could modulate ATG9A mRNA. The targeted binding sites of the ATG9A 3’-UTR were integrated into the pmirGLO dual-luciferase vector to produce the F-Luc reporter construct (Figure 1(b)). Second, HeLa and SiHa cells were co-transfected with mimics or mimics control, and the pmirGLO-ATG9A-Luc-WT or pmirGLO-ATG9A-Luc-MUT plasmid. The luciferase activity of CC cells co-transfected with mimics and the ATG9A-Luc-WT plasmid decreased significantly, while the reporter activity of CC cells co-transfected with the mimics control and ATG9A-Luc-WT plasmid or mimics control with pmirGLO-ATG9A-MuT did not change significantly, suggesting miR-195-5p abrogates the expression of ATG9A by directly targeting the ATG9A 3’-UTR in CC cells (Figure 1(c,d)).

The ability of miR-195-5p to regulate endogenous ATG9A protein was detected. miR-195-5p mRNA levels were enhanced remarkably by transfection with mimics in CC cells (Figure 1(e,h)). The mimics group showed significantly decreased expression of ATG9A protein (Figure 1(g–j)) as well as suppression of ATG9A mRNA (Figure 1(k,l)). By contrast, when miR-195-5p levels were reduced by transfection with inhibitor (Figure 1(e,f)), ATG9A protein (Figure 1(g-j)) and mRNA levels (Figure 1(k,l)) increased accordingly. Therefore, these results suggest miR-195-5p can produce a marked regulatory effect by inhibiting ATG9A mRNA and protein in CC cells.

### *Overexpression of miR-195-5p inhibited EMT* in vitro

**Figure 2.**
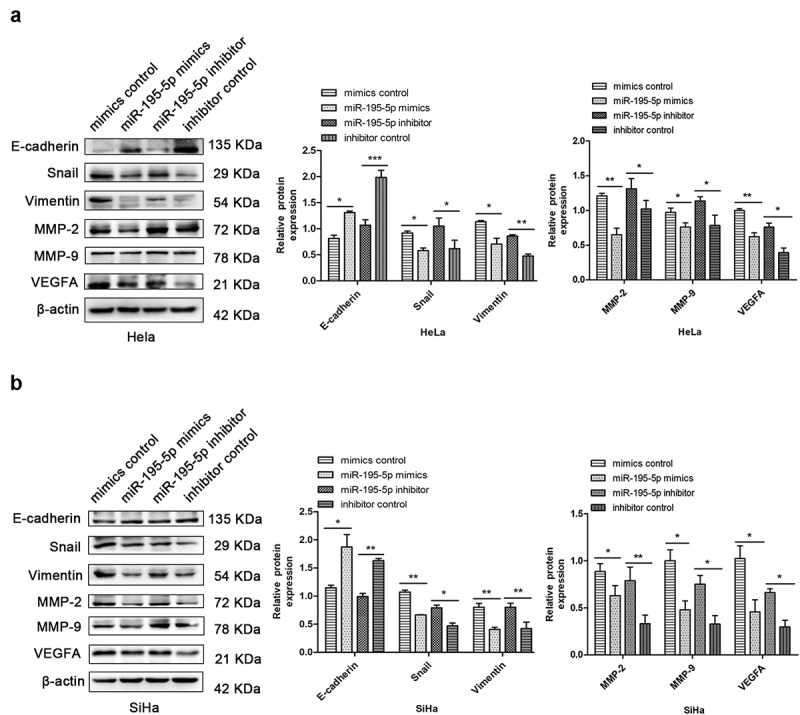
Upregulation of miR-195-5p represses EMT in CC cells. (a,b) western blotting showed that upregulation of miR-195-5p enhanced the expression levels of E-cadherin and decreased the expression levels of Snail, Vimentin, MMP-2, MMP-9, and VEGFA in HeLa and SiHa cells, respectively. Downregulation had opposite effects. β-actin was used as an internal control. Experiments were repeated three times (*n* = 3). Student’s *t*-tests were used as statistical analyses. *p < 0.05, **p < 0.01, and ***p < 0.001 versus control groups.

EMT is one of the most critical regulatory steps in malignant tumour metastasis. The deficiency of epithelial cells and gaining of mesenchymal cells make cancer cells more prone to metastasize [25]. Western blotting showed that upregulation of miR-195-5p dramatically increased expression of E-cadherin and reduced expression of Snail and Vimentin in HeLa and SiHa cells, respectively. miR-195-5p overexpression decreased the expression of matrix metalloproteinases MMP-2 and MMP-9, and decreased the expression of vascular endothelial growth factor VEGFA, a marker of angiogenesis and lymphangiogenesis (Figure 2(a,b)) in CC cells. Conversely, inhibition of miR-195-5p blocks EMT and lymphatic metastasis (Figure 2(a,b)). Thus, our findings show that overexpression of miR-195-5p can suppress the EMT and lymphatic vessel metastasis of CC cells, consistent with a previous study [13].

### Upregulation of miR-195-5p increased apoptosis and autophagy of CC cells

**Figure 3.**
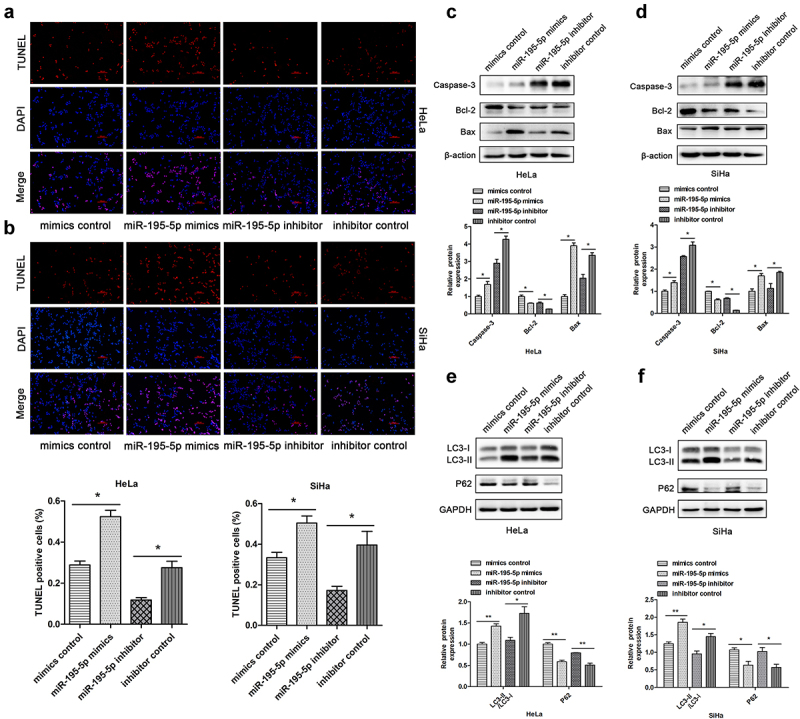
Overexpression of miR-195-5p induces apoptosis and autophagy of CC cells. HeLa and SiHa cells were transfected with mimics or inhibitor and negative control for 48 h. (a,b) TUNEL assay of fluorescence images of each group in HeLa and SiHa cells (scale bar, 100 μm). TUNEL staining analysis was used to detect apoptosis after transfection. The percentage of TUNEL-positive cells in each group is shown in the bar graph. (c,d) expression of Caspase-3, Bcl-2, and Bax measured by western blotting. (e,f) expression of LC3-II and P62 by western blotting. Data are expressed as mean ± SD. *p < 0.05, **p < 0.01, and ***p < 0.001 versus control group.

Next, the effect of miR-195-5p on autophagy and apoptosis of CC cells was characterized. TUNEL assays demonstrated that upregulation of miR-195-5p increased the apoptotic rate. Rather, miR-195-5p inhibition lessened cell apoptosis rate (Figure 3(a,b)). Subsequently, we assessed apoptosis-associated proteins by western blotting. The miR-195-5p mimics group significantly enhanced pro-apoptotic expression of Caspase-3 and Bax, but impaired expression of anti-apoptotic Bcl-2 compared with the mimics control group. Nevertheless, miR-195-5p inhibition suppressed the expression of Caspase-3 and Bax, but promoted the expression of Bcl-2 (Figure 3(c,d)). When autophagy is initiated, cytosolic LC3 enzymatically dissolves a small amount of polypeptide to form LC3-I, which binds PE and transforms it into a membrane type (i.e., LC3-II). Therefore, the expression level of LC3-II can be used to estimate the occurrence of autophagosomes [26]. In this experiment, we unexpectedly found that miR-195-5p could induce autophagy in CC cells. Western blot analysis demonstrated that overexpression of miR-195-5p increased the expression of LC3-II and suppressed expression of P62 (Figure 3(e,f)). By contrast, expression of LC3-II protein was dramatically reduced due to suppression of miR-195-5p, but P62 expression was increased (Figure 3(e,f)). In conclusion, overexpression of miR-195-5p could accelerate CC cell apoptosis and autophagy.

### ATG9A overturned repression of miR-195-5p in the malignant biological behaviour of CC cells

**Figure 4.**
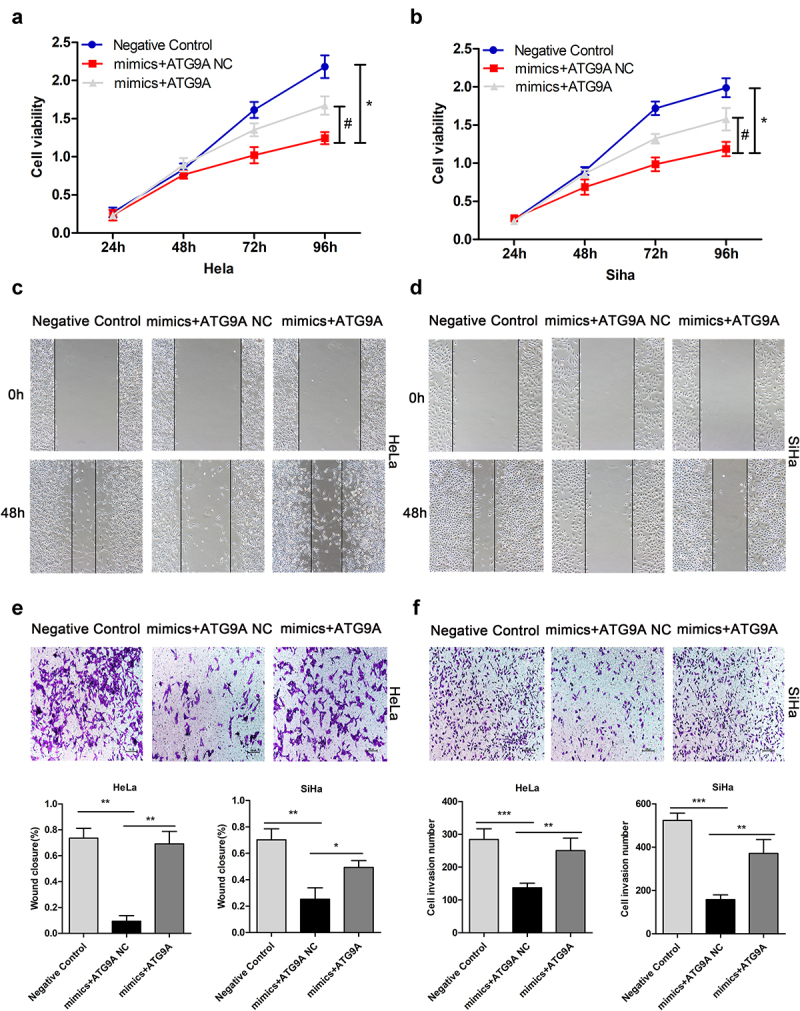
Upregulation of ATG9A partially overturned the effects of miR-195-5p on proliferation, migration, and invasiveness of CC cells. HeLa and SiHa cells were transfected with negative control, mimics + ATG9A NC, and mimics + ATG9A. (a,b) MTT assay was used to assess HeLa and SiHa cell proliferation. (c,d) wound healing assay was used to assess migration of CC cells following transfection (magnification, ×100). (e,f) Transwell assay (magnification, ×100) was performed to check invasiveness of CC cells. Experiments were repeated three times (*n* = 3) and quantification is shown in the bar graph. Student’s *t*-tests were used for statistical analyses. *p < 0.05, **p < 0.01, and ***p < 0.001 versus control group.

After determining the relationship between miR-195-5p and ATG9A, we turned out attention to the interaction of miR-195-5p and ATG9A and the malignant biological behaviour of CC cells, such as cell proliferation, migration, and invasiveness. We previously demonstrated that miR-195-5p could inhibit proliferation, migration, and invasion by targeting YAP1 in CC cells [13]. At present, there is no research to show whether it can inhibit its malignant biological behaviour by targeting ATG9A. To address this question, we first constructed an ATG9A overexpression plasmid and ATG9A negative control. Then, mimics negative control + ATG9A negative control (negative control), mimics + ATG9A negative control (mimics + ATG9A NC), and mimics + ATG9A were co-transfected into HeLa and SiHa cells. Then, MTT, wound healing, and Transwell assays were performed to assess CC cell proliferation, migration, and invasion, respectively. Compared with the mimics + ATG9A NC group, the mimics + ATG9A group significantly enhanced cell proliferation (Figure 4(a,b)). Consistently, overexpression of ATG9A overturned the inhibitory effects of miR-195-5p on migration (Figure 4(c,d)) and invasiveness (Figure 4(e,f)) of CC cells. Thus, miR-195-5p can restrain the malignant biological behaviour of CC cells by directly targeting ATG9A.

### ATG9A restored miR-195-5p-induced EMT in CC cells

**Figure 5.**
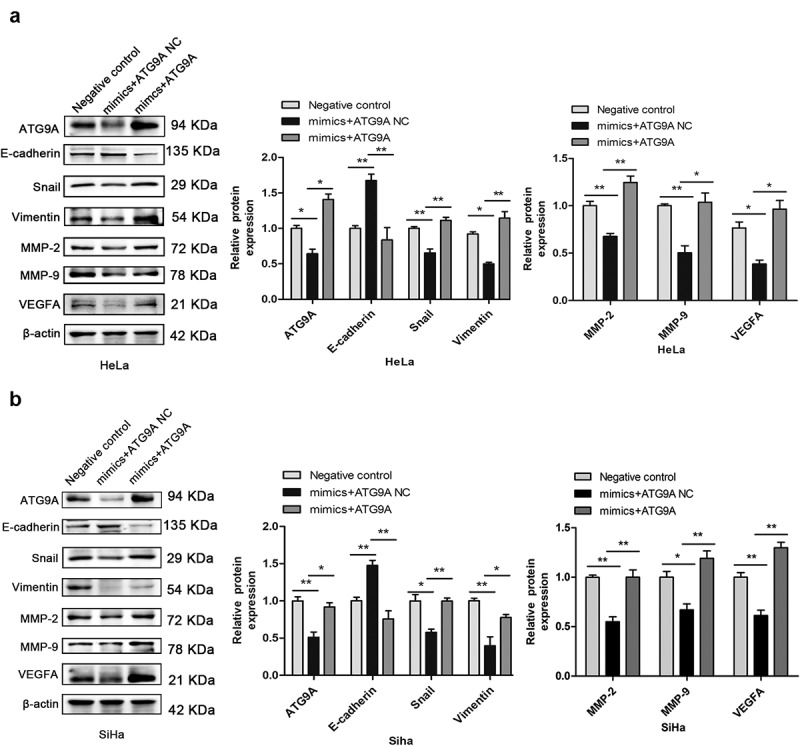
Overexpression of ATG9A partially rescued miR-195-5p-mediated inhibition of EMT. (a,b) western blotting was used to determine expression of ATG9A, E-cadherin, Vimentin, Snail, MMP-2, MMP-9, and VEGFA. Experiments were repeated three times (*n* = 3) and quantification is shown in the bar graph. Student’s *t*-tests were used for statistical analyses. *p < 0.05 and **p < 0.01 versus control group.

Following transfection, western blotting showed that expression of ATG9A protein was markedly increased in the mimics + ATG9A group compared with the mimics + ATG9A NC group. Overexpression of ATG9A significantly reversed EMT induced by miR-195-5p. As shown in Figure 5(a,b), overexpression of ATG9A significantly inhibited expression of E-cadherin, and restored expression of Snail, Vimentin, MMP-2, MMP-9, and VEGFA. These results suggest that the mechanism of regulation of EMT by miR-195-5p in CC cells is partly due to inhibition of ATG9A expression.

### *miR-195-5p promoted apoptosis and autophagy by inhibiting ATG9A* in vitro

**Figure 6.**
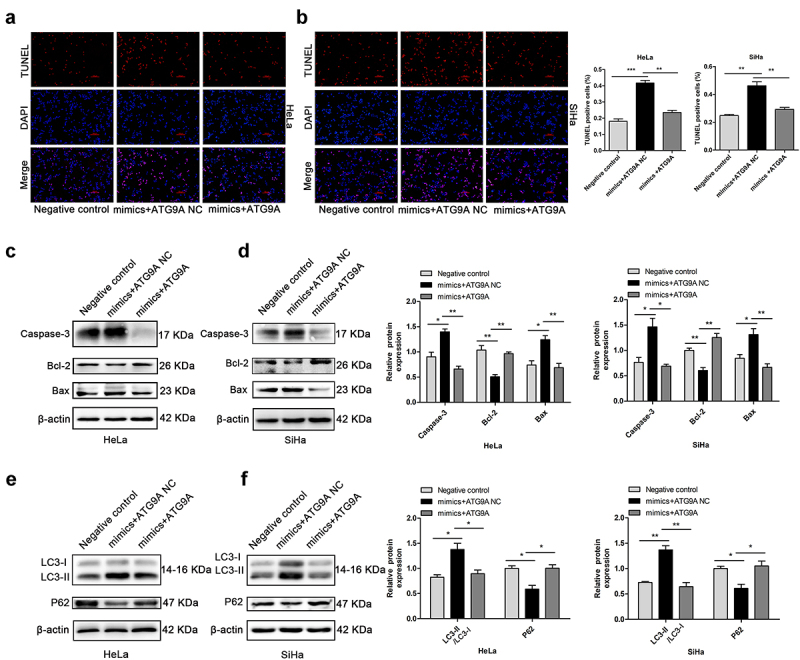
MiR-195-5p induces apoptosis and autophagy of CC cells by inhibiting ATG9A. (a,b) TUNEL assay (magnification, ×100) was performed after transfection with negative control, mimics + ATG9A NC, and mimics + ATG9A. (c,d) expression of Caspase-3, Bcl-2, and Bax measured by western blotting. (e,f) expression of LC3-II and P62 by western blotting. Experiments were repeated three times (*n* = 3). *p < 0.05 and **p < 0.01 versus control group.

To demonstrate whether miR-195-5p could regulate apoptosis and autophagy in CC cells by regulating ATG9A, a negative control, mimics + ATG9A NC, and mimics + ATG9A were co-transfected into HeLa and SiHa cells. TUNEL analysis showed that the rate of apoptosis of the mimics + ATG9A group was much lower than the mimics + ATG9A NC (Figure 6(a,b)). Consistently, western blotting showed that overexpression of ATG9A in the mimic + ATG9A group successfully suppressed expression of Caspase-3 and Bax and restored expression of Bcl-2 compared with the mimics + ATG9A NC group (Figure 6(c,d)). Furthermore, overexpression of ATG9A led a decrease in LC3-II protein expression and accumulation of P62, which partially restored the induction of autophagy by miR-195-5p (Figure 6(e,f)). Therefore, overexpression of ATG9A partially reversed apoptosis and autophagy induced by miR-195-5p.

## Discussion

Recurrence and metastasis of CC are key factors restricting the curing of this disease. It is important to determine the molecular mechanisms of CC metastasis and identify targeted treatments for patients with advanced CC. Accumulating evidence shows that miR-195-5p acts as a tumour suppressor gene in colorectal cancer [27], gastric cancer [28], lung adenocarcinoma [29], glioma [30], and CC [14,15]. Zhou et al. [15] and Wang et al. [14] showed that miR-195 was significantly reduced in CC tissues and cell lines, and the low miR-195 level was significantly correlated with higher International Federation of Gynecology and Obstetrics stage, node metastasis, and deep stromal invasion. Consistent with our research, upregulation of miR-195 suppressed cell migration and invasion in *vitro*. Why is miR-195-5p deregulated in CC? Current studies have demonstrated that DNA methylation is one of the earliest DNA modification pathways, and hypermethylation of CpG islands upstream of the promoter region of miRNA genes can lead to decreased expression, plays an important role in carcinogenesis [31–33]. In addition, persistent high-risk HPV infection was found to reduce cellular miRNA expression through the E6 viral oncoprotein [34]. Chromosome instability can change the expression pattern of miRNA [35]. Single nucleotide polymorphism (SNP) is DNA sequence polymorphisms at the genome level that are caused by single nucleotide variations. It has been found that SNP at miRNA binding sites can alter the function of messenger RNA target genes, this leads to cancer susceptibility and tumour progression [36]. Although miRNA is closely related to the occurrence and development of CC, the exact pathogenesis of miRNA is still not clear. A large number of miRNA studies are still needed to guide clinical practice and provide more objective and accurate molecular markers for the benefit of CC patients.

To date, many studies suggest a dual role for autophagy in cancer, depending on the type, stage, or genetic background of the cancer. Autophagy can both act as a tumour suppressor in the early stages of progression and promote tumorigenesis and induce resistance to therapeutic drugs in the late stages of progression [37]. Many miRNAs have been shown to be involved in autophagy. For example, miR-29a functions as a potent autophagy inhibitor in pancreatic ductal cell carcinoma and increases the sensitivity of cancer cells to chemotherapeutics [38]. Kwon et al. found that by directly targeting HDAC4, overexpression of miR-145-3p stimulated apoptosis in multiple myeloma cells and induced autophagy and cell death [7]. Zhu et al. [39] demonstrated that expression of P62 and Bcl-2 was upregulated, while expression of LC3BII/I, Bax, and cleaved Caspase-3 was downregulated after miR-195 was inhibited, indicating that low expression of miR-195 could attenuate neuroblastoma autophagy and apoptosis. In other words, miR-195 could induce autophagy and apoptosis, consistent with our findings. Our study first confirmed that miR-195-5p could induce autophagy in CC cells. However, the role of miR-195-5p in CC cell autophagy is not fully understood. Many studies have shown that EMT induces cancer initiation, including cancer invasiveness, therapeutic resistance, and tumour stemness [40,41]. Our study showed that miR-195-5p could not only inhibit EMT and expression levels of ATG9A in CC cells, but also induce autophagy and apoptosis. At the same time, ATG9A overexpression could reverse the inhibitory effect of miR-195-5p in CC. These results were consistent with previous studies showing miR-195-5p functions as a tumour suppressor gene. Although some potential targets of miR-195-5p have been reported, the molecular mechanism of miR-195 remains unknown. MiR-195-5p not only regulated invasiveness and metastasis of CC cells, but also promoted apoptosis and induced autophagy *in vitro*. These results suggest upregulation of miR-195-5p can prevent malignant progression of CC.

In this present study, we screened ATG9A for the first time as a miR-195-5p candidate target gene by bioinformatics analysis. Mammalian ATG9A is the only transmembrane protein in the core ATG protein, and its dysregulation is related to cancer [16]. A previous study has found that overexpression of ATG9A protein contributed to neoplasm volume enlargement and lymph node metastasis in oral squamous cell carcinoma. Also, Kaplan – Meier plots showed patients with ATG9A overexpression had shorter 3-year overall survival and time to recurrence [19]. Aurore et al. found an increase in ATG9A mRNA expression in TNBC samples, and that ATG9A inhibition resulted in suppression of cancer signatures in *vitro* [21]. Similarly, ATG9A, a downstream target gene of miR-29b, was significantly elevated in ovarian cancer, and ATG9A protein expression level was negatively correlated with overall survival and progression-free survival [20]. These results are in agreement with our conclusion that overexpression of ATG9A partially relieved the inhibition of miR-195-5p on CC cell proliferation, migration, invasiveness, EMT, and lymph node metastasis. Furthermore, overexpression of ATG9A reversed apoptosis and autophagy mediated by miR-195-5p *in vitro*. It is worth noting that ATG9A acts as an autophagy-related protein, and its depletion inhibits autophagy. Contrary to our findings, Pang et al. found that overexpression of miR-34a significantly reduced the expression level of ATG9A in the inner ear HEI-OC1 cell line, while knockdown of ATG9A inhibited autophagic flux [22], while our conclusions show that upregulation of miR-195-5p induces autophagy and overexpression of ATG9A partially reverses miR-195-5p-induced autophagy. Orsi et al. found that mammalian Atg9 (mAtg9) is essential for the initiation and progression of autophagy. However, it does not act as a structural component of the autophagosome; it is required for the expansion of autophagosome precursors. Additionally, small amounts of autophagosomes were able to form even in the absence of mAtg9, suggesting mAtg9 is not strictly required for autophagosome formation [42]. Whether miR-195-5p induces autophagy in CC cells via ATG9A remains unknown. Future studies will need to explore the relationship between other targets of miR-195-5p and autophagy in CC cells and related factors affecting autophagy.

## Conclusions

Our study determined the inhibitory roles of miR-195-5p on CC cell proliferation, migration, invasion, and EMT, as well as the promotion of autophagy and apoptosis *in vitro*. These effects were partly due direct targeting of ATG9A in the progression of CC, which may give novel insight into tumour development, and may be clinically useful as a biomarker for the treatment of CC. The miR-195-5p/ATG9A axis may be a molecular target for the advancement of CC treatment technology. There are some limitations of our study, including the lack of validation of these experimental xenograft models to visualize tumour progression in a more complex system. Non-coding RNA and autophagy processes remain an attractive target for therapy. Future studies are needed to identify new miRNA molecules and upstream regulatory molecules with special value for gene therapy and CC diagnosis. A large number of *in vivo* experiments will also be required to verify the specific effects of autophagy in different phases of cancer progression, and to find better treatment options for patients with advanced CC.

## Highlights

- Upregulation of miR-195-5p not only inhibits proliferation, migration, and the EMT of CC cells, but also induces apoptosis and autophagy.
- MiR-195-5p targeted and negatively regulated ATG9A.
- Rescue experiments suggested that overexpression of ATG9A could partially abolish miR-195-5p-mediated suppression of CC cells.

## Data Availability

All supporting data are included within the main article.
